# Late intrathoracic migration of a retained temporary epicardial pacing wire associated with middle lobe atelectasis and hemoptysis 15 years after aortic valve replacement: a case report

**DOI:** 10.1186/s44215-026-00274-1

**Published:** 2026-08-25

**Authors:** Shiro Kaneda, So Miyahara, Shohei Mitsumata, Jyun-ichi Wakahara, Kensuke Midorikawa, Yuichiro Ueda, Toshihiko Sato

**Affiliations:** Department of General Thoracic, Breast, and Pediatric Surgery, Fukuoka University School of Medicine and Hospital, 7-45-1 Nanakuma, Jonan-Ku, Fukuoka City, Fukuoka, 814-0180 Japan

**Keywords:** Intrathoracic migration, Temporary epicardial pacing wire, Atelectasis, Hemoptysis

## Abstract

**Background:**

Temporary epicardial pacing wires (TEPWs) are routinely placed after cardiac surgery for temporary rhythm management and are typically removed within a few days. Although retained wires are uncommon, late migration may lead to serious complications.

**Case presentation:**

An 80-year-old man who had undergone aortic valve replacement 15 years earlier was referred for evaluation of right middle lobe atelectasis detected on computed tomography (CT). Bronchoscopy revealed mucus obstruction and distal mucosal edema of the right middle lobe bronchus. The patient subsequently developed hemoptysis. Contrast-enhanced CT demonstrated a continuous 11-cm linear high-density structure extending from the right hilum to the inferior cardiac surface, near the middle lobe bronchovascular structures. Intrathoracic migration of a retained TEPW was suspected, and surgical removal was performed. Thoracoscopic exploration revealed dense hilar adhesions; therefore, the pericardium was opened through the same thoracoscopic ports, and the wire was removed using an intrapericardial approach. The postoperative course was uneventful.

**Conclusions:**

Retained TEPWs may migrate intrathoracically even many years after cardiac surgery. In the present case, the migrated wire was anatomically adjacent to the middle lobe bronchovascular structures and was clinically associated with atelectasis and hemoptysis, although direct bronchial penetration was not confirmed. In selected cases with hilar or pericardial-adjacent migration, an intrapericardial approach may facilitate safe removal.

## Background

Temporary epicardial pacing wires (TEPWs) are routinely placed after cardiac surgery and are generally removed within a few days [1]. Although retained TEPWs are uncommon, late migration-related complications have been reported [2]. We report a rare case of late intrathoracic migration of a retained TEPW associated with right middle lobe atelectasis and hemoptysis 15 years after aortic valve replacement.

## Case presentation

**Fig. 1:**
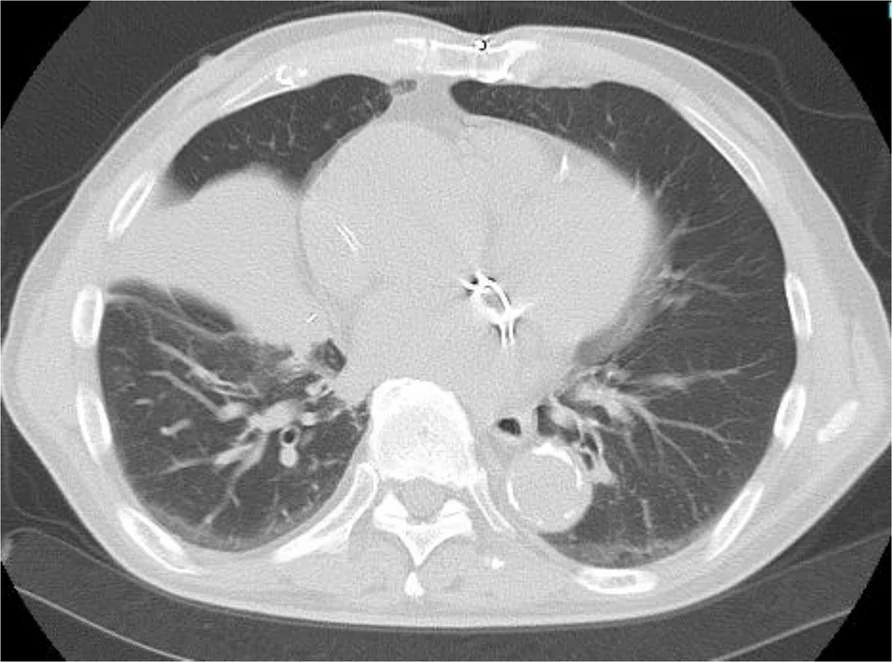
Chest computed tomography showing right middle lobe atelectasis

**Fig. 2:**
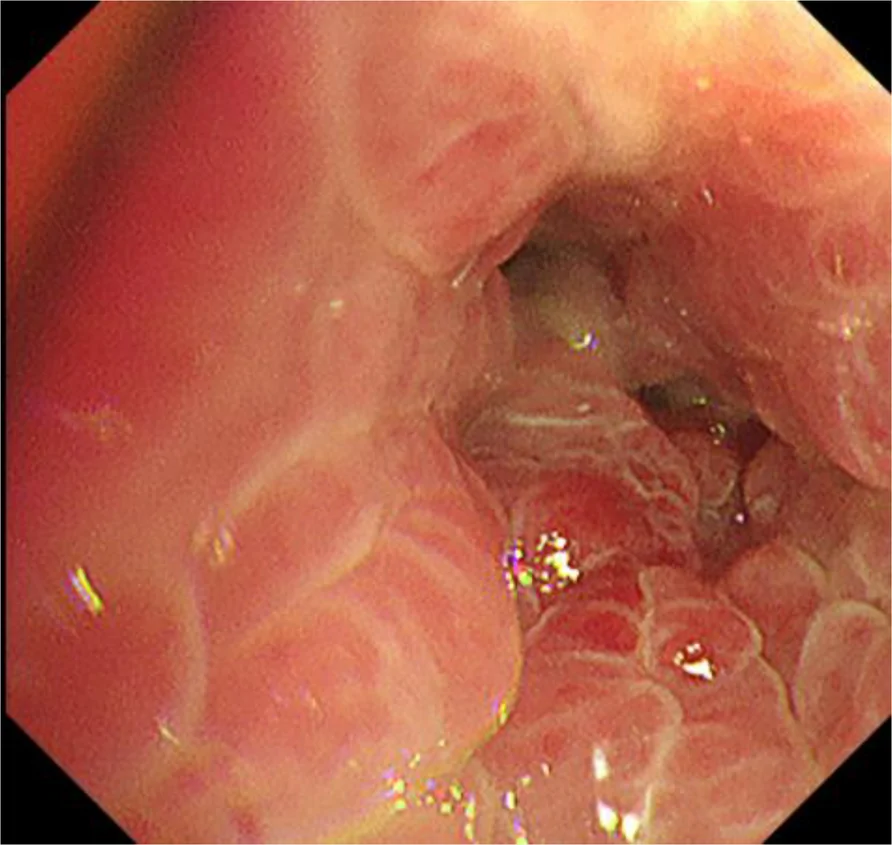
Bronchoscopic image showing mucus impaction and distal mucosal edema in the right middle lobe bronchus

**Fig. 3:**
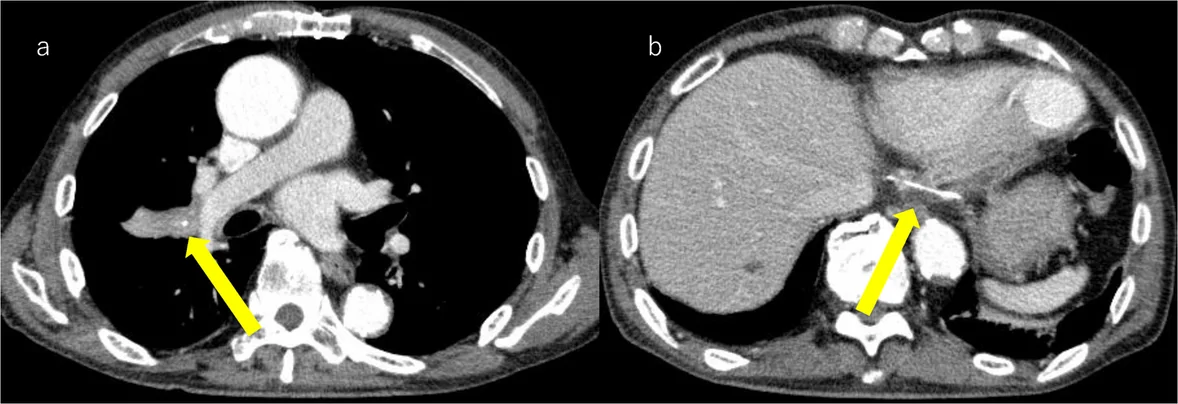
**a** Contrast-enhanced axial chest computed tomography showing a linear high-density structure in the right hilum (arrow). **b** Continuity of the same structure extending toward the inferior aspect of the heart (arrow)

**Fig. 4:**
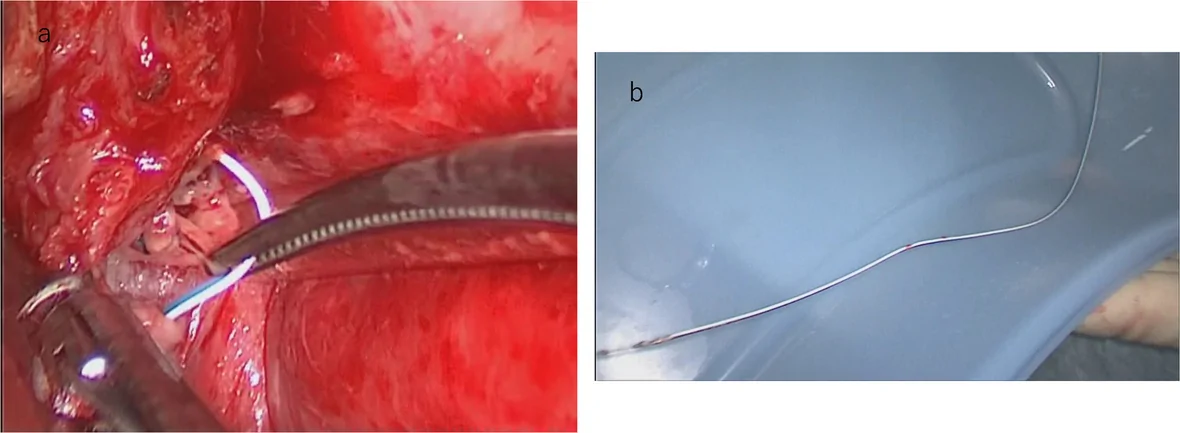
**a** Thoracoscopic retrieval of the retained temporary epicardial pacing wire via the intrapericardial approach. **b** Extracted temporary epicardial pacing wire

An 80-year-old man had undergone mitral valve plasty and aortic valve replacement with a bioprosthetic valve for mitral regurgitation and severe aortic regurgitation 15 years previously. A TEPW was placed at that time. The postoperative record indicated that resistance was encountered during attempted removal of the TEPW; therefore, the wire was transected, with a portion intentionally left in situ. During preoperative evaluation for cervical spondylotic myelopathy, chest CT incidentally revealed right middle lobe atelectasis (Fig. 1). The patient was referred to our department for further evaluation. Bronchoscopy demonstrated mucus obstruction at the orifice of the right middle lobe bronchus and edematous narrowing of the distal mucosa (Fig. 2). As a result, the distal bronchial lumen could not be visualized bronchoscopically. Initially, conservative management was adopted. However, the patient subsequently developed hemoptysis. Contrast-enhanced chest CT revealed a continuous, approximately 11-cm linear high-density structure extending from the right hilar region to the inferior cardiac surface (Fig. 3a, b). The distal portion of the structure was located in the right hilar region, adjacent to the middle lobe bronchovascular structures, and appeared to course near the inferior pulmonary vein. There was no clear radiological evidence of intraluminal bronchial penetration, intravascular migration into the pulmonary vessels, contrast extravasation, or pseudoaneurysm. Based on the imaging findings and surgical history, intrathoracic migration of a retained TEPW was suspected to be associated with the middle lobe atelectasis and hemoptysis. Because malignant bronchial obstruction could not be completely excluded based on the initial bronchoscopic findings, bronchoscopic biopsy of the abnormal bronchial mucosa was performed on the day of surgery before thoracoscopic removal. Frozen-section analysis showed no evidence of malignancy. The operation was initiated using a 3-port VATS approach without median sternotomy or thoracotomy. Dense hilar adhesions, particularly around the pulmonary veins, made pleural-side dissection unsafe. Therefore, through the same thoracoscopic ports, the pericardium was opened anterior to the phrenic nerve in collaboration with cardiac surgeons. Intrapericardial dissection was then performed from the basal aspect of the heart toward the posterior pericardial space. The intrapericardial portion of the retained TEPW was identified near the inferior pulmonary vein and removed without bleeding or air leakage (Fig. 4a, b). No bronchial fistula, purulent fluid, or macroscopic evidence of infection was observed. Bacterial culture of the removed wire was not performed.

**Fig. 5:**
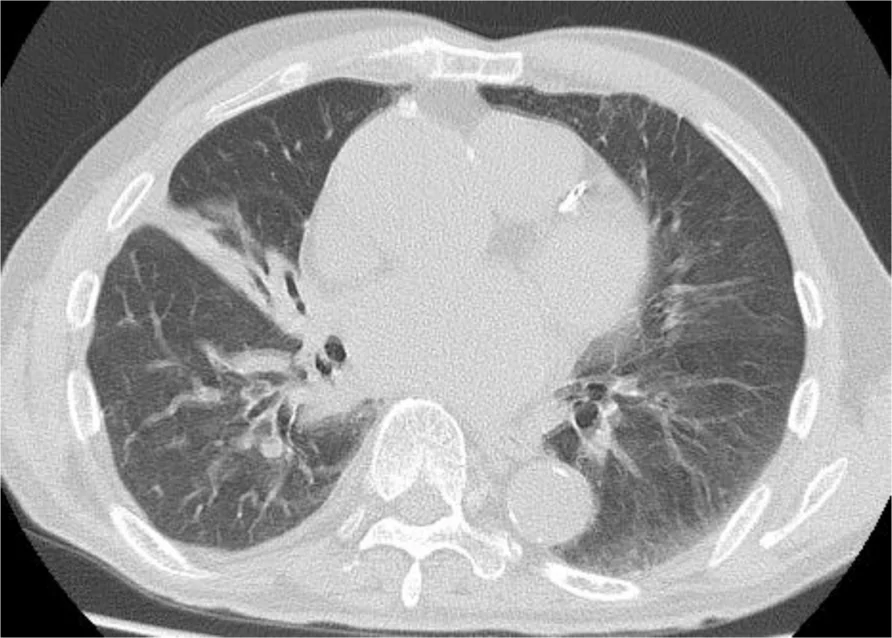
Chest computed tomography obtained 8 months after surgery showing resolution of right middle lobe atelectasis

The postoperative course was uneventful. At 8-month follow-up, atelectasis had improved and hemoptysis had not recurred (Fig. 5).

## Discussion

Late complications related to retained temporary epicardial pacing wires (TEPWs) are uncommon but clinically important [1]. When resistance is encountered, the wire may be cut flush with the skin and left in situ, although the long-term safety of this practice remains controversial [3]. No standardized long-term follow-up protocol has been established; however, documentation, patient notification, and clinical awareness of retained TEPWs as a possible cause of unexplained thoracic, respiratory, infectious, or cardiovascular symptoms are important. In the present case, the exact timing of extrapericardial migration could not be determined. Retrospective review showed that the linear high-density structure was already present on the initial CT, whereas the retained wire was difficult to identify on chest radiography because of overlap with the mediastinal and hilar structures. Because no interval chest CT images were available between the index cardiac operation and the current presentation, gradual migration over several years could not be excluded.

Table 1 summarizes previously reported complications of retained TEPWs, including cardiovascular migration [4–6], intrathoracic migration into the chest wall or lung [2, 7], and bronchial penetration [8]. Removal approaches have included transcatheter, transbronchial, and thoracic procedures according to the migration site.

**Table 1:** Summary of previously reported complications associated with retained temporary epicardial pacing wires (TEPW)

Author (year)	Interval from cardiac surgery	Presenting symptoms	Migration site	Retrieval method	Key clinical feature
Present case	15 years	Middle lobe atelectasis → hemoptysis	Right hilum to inferior cardiac surface (no direct perforation)	VATS + Intrapericardial approach	Inflammatory bronchial obstruction without penetration; required intrapericardial exposure
Polomsky et al. (2020) [2]	18 months	Hemoptysis, empyema	Chest wall/perithoracic migration	Thoracotomy	Epicardial pacemaker lead rather than temporary pacing wire
Worth et al. (2011) [4]	24 years	Incidental finding/asymptomatic	Pulmonary artery	Percutaneous catheter-based retrieval	Right ventricular to pulmonary artery migration
Wolf et al. (2013) [5]	9 months	Infection	Ascending aorta	Percutaneous catheter-based retrieval	Intra-aortic migration
Meier et al. (2004) [6]	3 years	Ventricular tachycardia, cardiac arrest	Right atrium → right ventricle → pulmonary artery	Endovascular snare retrieval	Intravascular migration causing arrhythmia
Horng et al. (2008) [7]	6 years	Progressive dyspnea, recurrent pneumonia	Right upper lobe pulmonary parenchyma	Thoracotomy	Parenchymal migration with bronchiectasis
Roy et al. (2022) [8]	13 months	Airway symptoms	Bronchial penetration	Bronchoscopic extraction	Direct bronchial perforation

Reported cases of retained temporary epicardial pacing wire migration and comparison with the present case. The present case was characterized by late intrathoracic migration causing lobar atelectasis and requiring an intrapericardial approach for removal

In the present case, contrast-enhanced CT suggested that the retained TEPW was located near the inferior pulmonary vein and middle lobe bronchovascular structures, indicating a hilar and pericardial-adjacent location rather than definite intrabronchial or intravascular migration. Based on the CT course and intraoperative findings, the migrating end was considered to be the transected cutaneous end, whereas the cardiac end was presumed to have remained near the inferior cardiac surface, possibly on the inferior, or diaphragmatic, aspect of the right ventricle. However, the initial record did not document the exact placement site or whether the cardiac end had been additionally suture-fixed to the epicardium. Although intravascular migration, contrast extravasation, and pseudoaneurysm were not evident, vascular injury remained a concern because of the proximity to the pulmonary veins and hilar vessels. Therefore, the operation was performed in collaboration with cardiac surgeons. When dense hilar adhesions precluded pleural-side dissection, an intrapericardial approach enabled thoracoscopic identification and controlled removal of the wire. Because direct bronchial penetration was not confirmed, bronchial edema and atelectasis may have resulted from indirect mechanisms, such as inflammatory bronchial narrowing, peribronchial irritation, or extrinsic compression, possibly related to a foreign-body reaction to the retained wire. The absence of air leakage or bleeding after wire removal, together with subsequent improvement of atelectasis and no recurrence of hemoptysis, was consistent with this interpretation. This approach may be useful for retained TEPWs near the hilum or pericardium, particularly when vascular injury is a concern.

## Data Availability

Not applicable.

## Abbreviations

CT: Computed tomography
TEPWs: Temporary epicardial pacing wires
VATS: Video-assisted thoracic surgery
